# Carbazole hydroxylation by the filamentous fungi of the *Cunninghamella* species

**DOI:** 10.1007/s11356-015-5146-7

**Published:** 2015-08-16

**Authors:** K. Zawadzka, P. Bernat, A. Felczak, K. Lisowska

**Affiliations:** Department of Industrial Microbiology and Biotechnology, Faculty of Biology and Environmental Protection, University of Lodz, 12/16 Banacha Street, 90-237 Lodz, Poland

**Keywords:** Carbazole, Degradation, Phospholipids, N-heterocycles, Detoxification

## Abstract

Nitrogen heterocyclic compounds, especially carbazole, quinolone, and pyridine are common types of environmental pollutants. Carbazole has a toxic influence on living organisms, and the knowledge of its persistence and bioconversion in ecosystems is still not complete. There is an increasing interest in detoxification of hazardous xenobiotics by microorganisms. In this study, the ability of three filamentous fungi of the *Cunninghamella* species to eliminate carbazole was evaluated. The *Cunninghamella elegans* IM 1785/21Gp and *Cunninghamella echinulata* IM 2611 strains efficiently removed carbazole. The IM 1785/21Gp and IM 2611 strains converted 93 and 82 % of the initial concentration of the xenobiotic (200 mg L^−1^) after 120 h incubation. 2-Hydroxycarbazole was for the first time identified as a carbazole metabolite formed by the filamentous fungi of the *Cunninghamella* species. There was no increase in the toxicity of the postculture extracts toward *Artemia franciscana*. Moreover, we showed an influence of carbazole on the phospholipid composition of the cells of the tested filamentous fungi, which indicated its harmful effect on the fungal cell membrane. The most significant modification of phospholipid levels after the cultivation of filamentous fungi with the addition of carbazole was showed for IM 1785/21Gp strain.

## Introduction

Nitrogen heterocyclic aromatic compounds are one of the most important classes of the environmental pollutants, which have been detected in air, soil, and water samples (Singh et al. 2011a, b; Xu et al. 2006). Besides quinolone and pyridine, carbazole is a common contaminant generated from petrochemical and coking industries (Zhao et al. 2011b). This compound contaminates shale oil, crude oil, coal tar, and petroleum distillates (Castorena et al. 2006). Carbazole and its derivatives are used for the production of dyes, plastics, insecticides, and medicines (Loh and Yu 2000; Lobastova et al. 2004).

The mutagenic, carcinogenic, and toxic activities of carbazole have been documented (Jha and Bharti 2002; Jha et al. 2002; Mumbo et al. 2015). Its mutagenic potential toward Swiss albino mice was reveled in many studies. Carbazole induced dominant lethal mutation and formation of sperm head abnormalities in male germ cells of mice (Jha and Bharti 2002). Moreover, Jha et al. (2002) observed a clastogenicity effect of carbazole in the concentrations of 150 and 200 mg/kg body weight in Swiss albino mice. These concentrations of the xenobiotic caused a decrease in the mitotic index and an increase in the chromosomal aberration frequency in bone marrow cells (Jha et al. 2002). Mumbo et al. evidenced dioxine-like toxicity of carbazole in soil (Mumbo et al. 2015). According to the recommendation of the European Trade Union Institute (2009), carbazole was classified as a possible carcinogen to human (B2 group) and the Agency for Toxic Substances and Disease Registry (2015) included it in the priority list of hazardous substances.

The hydrophobic character of the xenobiotic promotes its accumulation in sediment, which may result in its long lasting harmful influence on aquatic organisms (Singh et al. 2011b; Zhao et al. 2011; Loh and Yu 2000). Microbial degradation can be an efficient method for the conversion of dangerous xenobiotics; therefore, a search for microorganisms capable of their utilization and finding the mechanisms of these processes are very important (Xu et al. 2006). Literature data point to many carbazole-degrading bacterial strains. Ouchiyama et al. (1993) isolated two bacterial strains of *Pseudomonas* sp., which have an ability to use carbazole as a sole source of carbon, nitrogen, and energy. The authors identified the main products of bacterial metabolism of carbazole and proposed a pathway of its biodegradation (Ouchiyama et al. 1993). The results of subsequent studies confirmed the pathway of bacterial degradation of carbazole, which had been suggested by Ouchiyama (Nam et al. 2012; Kirimura et al. 1999; Seo et al 2006). Carbazole 1,9a-dioxygenase (CARDO) is a key factor of bacterial metabolism of the xenobiotic. The first step of the catalysis by this enzyme is introducing two hydroxyl groups to the structure of carbazole and cleavage of its aromatic ring followed by a formation of 2′-aminobiphenyl-2,3-diol (Xu et al. 2006). The pathway of carbazole degradation by different bacteria and its molecular basis has been well documented. However, there are a few information about degradation of carbazole and its derivatives by filamentous fungi (Yang and Davis 1992, Lobastova at al. 2004; Parshikov et al. 2012). Filamentous fungi from the genus *Cunninghamella* demonstrated the capability of degrading a broad spectrum of xenobiotics (Lisowska and Długoński 2003; Bernat et al. 2013; Asha and Vidyavathi 2009; Parshikov et al. 2012).

The contact of microorganism cells with toxic and harmful substances can result in the perturbation of their structure and functionality. For this reason, bacteria and fungi have an ability to adapt and survive in the presence of xenobiotics (Dercová et al. 2004). Microorganisms can change the lipid molecular species proportion in response to a toxic compound. The use of liquid chromatography and tandem mass spectrometry (LC-MS/MS) techniques allows analyzing the lipid profile and its modification under the influence of stress factors (Welti et al. 2002).

The aim of this paper was to investigate the ability of carbazole degradation by three filamentous fungi from the genus *Cunninghamella* (*C. elegans* IM 1785/21Gp, *C. echinulata* IM 2611, *C. elegans* DSM 8217). We also analyzed the metabolites of carbazole produced by the tested microorganisms and their toxicity toward *Artemia franciscana.* Moreover, the modifications in the phospholipid profile of the filamentous fungi in response to carbazole were examined.

## Materials and methods

### Chemicals

Carbazole, 2-hydroxycarbazole, 4-hydroxycarbazole, and phenylcarbazole were purchased from Sigma. Other reagents were high purity grade chemicals obtained from POCH (Poland) and JT Baker (USA).

### Microorganisms and growth conditions

The *Cunninghamella elegans* IM 1785/21Gp and *Cunninghamella echinulata* IM 2611 came from the collection of the Department of Industrial Microbiology and Biotechnology, University of Lodz (Poland). *C. elegans* DSM 8217 was purchased from DSMZ collection (Germany). Spores of tested filamentous fungi from 7-day-old cultures were employed for the preparation of precultures in Sabouraud dextrose broth liquid medium (Difco, USA) supplemented with 2 % glucose. Microorganisms were cultured for 24 h at 28 °C on a rotary shaker (145 rpm) in 100 mL Erlenmeyer flasks. The precultures were resuspended in 40 mL fresh Sabouraud medium with 2 % glucose and cultivated for subsequent 24 h. The carbazole stock was prepared in a mixture of DMSO:Tween 80 (1:2). Carbazole elimination, dry weight, and lipid profile modification were examined in Czapek-Dox liquid medium, composed of 3 g L^−1^ NaNO_3_; 1 g L^−1^ KH_2_PO_4_; 0.5 g L^−1^ KCl; 0.5 g L^−1^ MgSO_4_ × 7 H_2_O; 0.01 g L^−1^ FeSO_4_ × 7 H_2_O; 40 g L^−1^ glucose. Erlenmeyer flasks (100 mL) containing 18 mL Czapek-Dox medium were supplemented with 200 mg L^−1^ carbazole and inoculated with 2 mL of previously prepared fungal biomass. Biotic controls were performed without the addition of carbazole. The cultures were carried out on a rotary shaker at 28 °C for 5 days. Each measured time point was prepared in three independent repetitions. For dry weight determination, the mycelium was separated by filtration, washed with distilled water, and dried at 100 °C to a constant weight.

### Carbazole extraction and GC-MS-analysis

After incubation, the filamentous fungi cultures were disintegrated for 5 min using Mixer Mill MM400 (Retsch, Germany) and 1 M HCl was added to pH 3. Carbazole and its metabolites were extracted with ethyl acetate (1:1, *v*/*v*). Ethyl acetate extracts were dehydrated using anhydrous sodium sulfate and then evaporated to dryness at 43 °C. After evaporation, each sample was resuspended in 2 mL ethyl acetate and analyzed by GC-MS. Phenylcarbazole was used as an internal standard in the quantitative analysis. The Agilent Technologies 7890A Gas Chromatography system equipped with a methyl polysiloxane HP 5MS column (30 m × 0.25 mm) and a 5975C Triple-Axis Detector (Agilent Technologies, USA) was used. The analysis was carried out using helium as a gas carrier at 1.2 mL min^−1^. In quantitative and qualitative methods, the oven temperature was set in the ranges of 100–300 and 80–300 °C, respectively. For the quantitative analysis, the ion at 167 m/z was used. The qualitative analyses were carried out at the mass range 100-400 m/z.

### Toxicity study

Toxicity of postculture extracts was evaluated using the Artoxkit M test (Microbiotests, Inc., Mariakerke-Gent, Belgium). Extracts of tested filamentous fungi incubated with carbazole and abiotic control were prepared according to p. 2.2. and 2.3. Brine shrimp cysts of *A. franciscana* were transferred to saline water in petri dishes and incubated for 48 h with an exposure to a light source at 3000 lux. The larvae of *A. franciscana* were incubated with postculture extracts or carbazole dissolved in DMSO. Toxicity of carbazole and its metabolites was calculated as a percentage of larvae that were not mobile after 48 h incubation.

### Lipid determination by HPLC-MS/MS analysis

Lipid profile determination was prepared according to Bernat et al. 2014 using the HPLC-MS/MS techniques. The mycelia of all tested fungi in the stationary phase of growth were separated from medium and crushed with 10 mL MeOH using a Mixer Mill MM400 (Retsch, Germany). The suspension was centrifuged at 6000 rpm for 3 min at 4 °C. The supernatant was isolated from biomass and vortexed for 2 min with an addition of 20 mL chloroform. Collected organic phases were anhydrated with sodium sulfate and evaporated to dryness. Extracts were redisolved in 500 μL of chloroform and suspended 25-fold in MeOH. An Agilent 1200 HPLC system and a 3200 Q-Trap mass spectrometer with ESI source were used for the analysis of polar lipids. A Synergi^TM^ Max-RP capillary column (50 mm × 2 mm, 4 μm particle size, 80 Å pore size, Phenomenex, Torrance, USA) was used for the reversed-phase chromatographic analysis. The temperature of the column was 40 °C. The injection volume was 5 μL. The 5 mM ammonium formate in water (A) and 5 mM ammonium formate in MeOH (B) were used as mobile phases. The solvent gradient with a constant flow rate of 600 μL min^−1^ was started at 60 % of eluent B and maintained to 0.5 min; 100 % of eluent B in 1.5 min and maintained to 6.5 min; and returned to the initial conditions in 8.1 min and maintained till 10 min for the stabilization of the column. The parameters of the ESI ion source were set as IS:−4500 V, CUR:25, GS1:50, GS2:60, and TEM:600 °C. The Analyst^TM^ version 1.5.1 software (AB Sciex, Framingham, MA, USA) was used in quantitative and qualitative analyses.

### Statistical analysis

All obtained results were presented as mean ± standard deviation (SD). The one-way analysis of variance (ANOVA) with *p* value <0.05 was used for the evaluation of statistical significance.

## Results and discussion

### Growth of the *Cunninghamella* strains with carbazole

Many *Cunninghamella* strains are able to metabolize different hazardous pollutants, including phenanthrene, fluorine, 1-methylnaphthalene, benzo[e]pyrene, malachite green, and 6-nitrochrysene. Moreover, these microorganisms can lead to the formation of heteroatom oxidized, N- or O-demethylated and hydroxylated metabolites (Lisowska and Długoński 2003; Črešnar and Petrič 2011; Zhang et al. 1996). Data concerning the pathway of carbazole bioconversion by filamentous fungi are still very limited in contrast to the information documenting the degradation ability of different bacteria (Zhao et al. 2011; Loh and Yu 2000; Larentis et al. 2011; Yoon et al. 2002). For this reasons we studied ability to carbazole degradation by *Cunninghamella* strains.

**Fig. 1:**
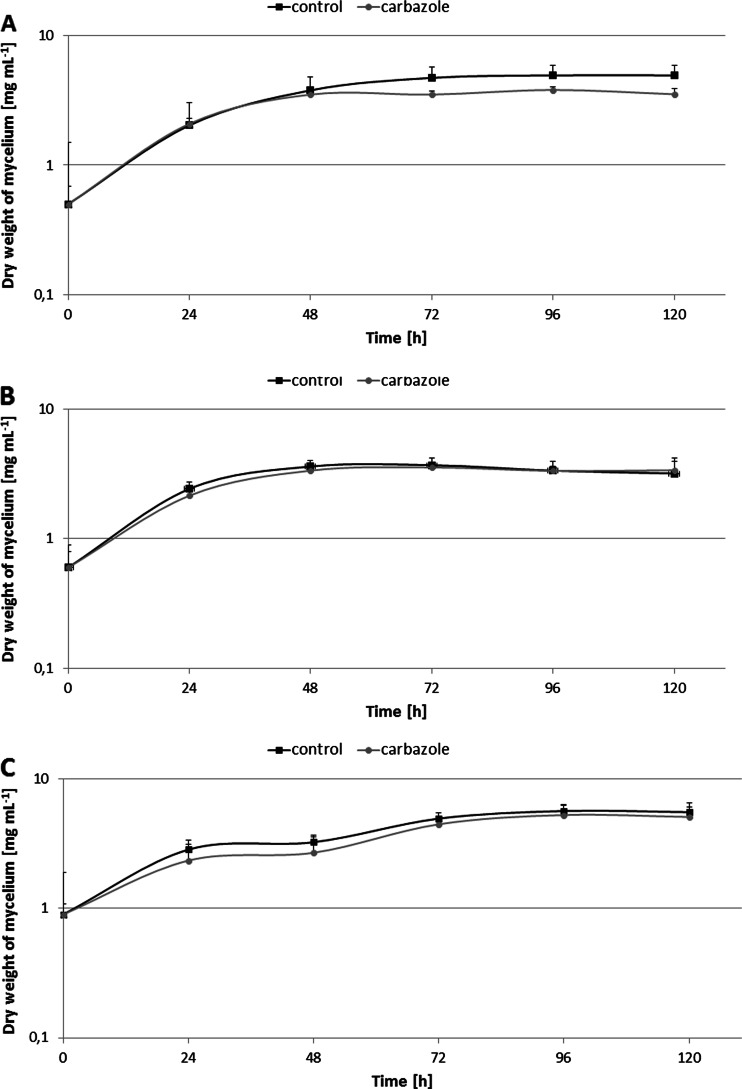
Growth of *C. elegans* IM 1785/21Gp (**a**), *C. echinulata* IM 2611 (**b**), and *C. elegans* DSM 8217 (**c**) in the presence of carbazole at the concentration 200 mg L^−1^ on Czapek-Dox liquid medium, during 120 h of incubation. Each result represents an average and SD was taken from *n* ≥ 3

In the present study, the influence of carbazole at the concentration of 200 mg L^−1^ on the growth of the filamentous fungi *C. elegans* IM 1785/21Gp, *C. echinulata* IM 2611, and *C. elegans* DSM 8217 was investigated (Fig. 1). All tested microorganisms showed high tolerance toward high concentrations of carbazole. The xenobiotic exhibited the highest toxicity toward the IM 1785/21Gp strain (Fig. 1a). In the cultures of this fungus, the 30 % limitation of growth compared with control samples without carbazole was noted after 120 h incubation. In the case of the DSM 8217 strain, an inconsiderable inhibition of growth was observed (Fig. 1c). Addition of carbazole to culture IM 2611 did not wield toxicity influence to growth of biomass synthesis (Fig. 1b).

### Quantitative and qualitative GC-MS analysis of carbazole elimination

**Fig. 2:**
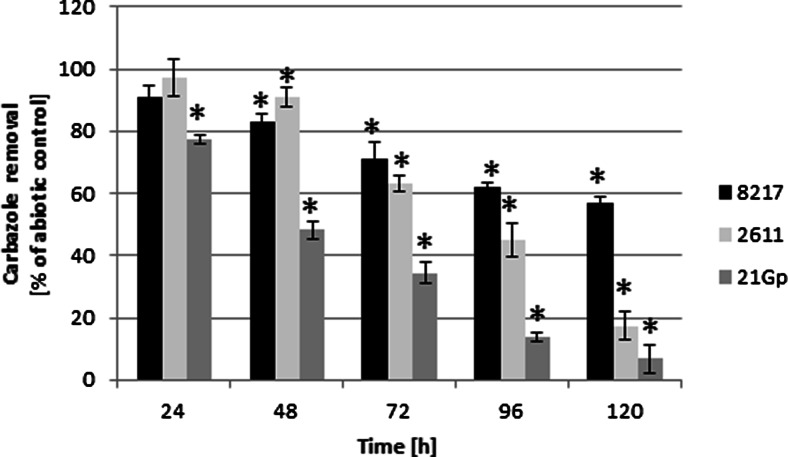
Elimination of carbazole (200 mg L^−1^) by tested microorganisms. Each result represents an average (*n* ≥ 3 collected from three independent experiments). Statistical analysis was done using the Student’s test with *p < 0.005

Samples for quantitative and qualitative analyses were collected after 24, 48, 72, 96, and 120 h cultivation of the filamentous fungi with carbazole. Reference samples were biotic and abiotic controls incubated for the same time periods. The ability of all the three *Cunninghamella* strains to utilize carbazole was demonstrated. The dynamics of carbazole elimination by the tested filamentous fungi is shown in Fig. 2. The fungus *C. elegans* IM 1785/21Gp was the most effective and eliminated almost completely 200 mg L^−1^ of carbazole within 120 h of incubation. High correlation between reduction of IM 1785/21Gp strain growth and its ability to carbazole elimination confirmed toxic influence of tested xenobiotic. *C. echinulata* IM 2611 also showed high effectiveness in removing the tested compound. The lowest capacity of carbazole biodegradation was reported for the *C. elegans* DSM 8217 strain. After 120 h cultivation, the filamentous fungi IM 1785/21Gp and IM 2611 with the addition of carbazole removed 93.1 and 82.6 % of the initial concentration of the xenobiotic, while the DSM 8217 strain eliminated only 43.1 % of carbazole.

**Fig. 3:**
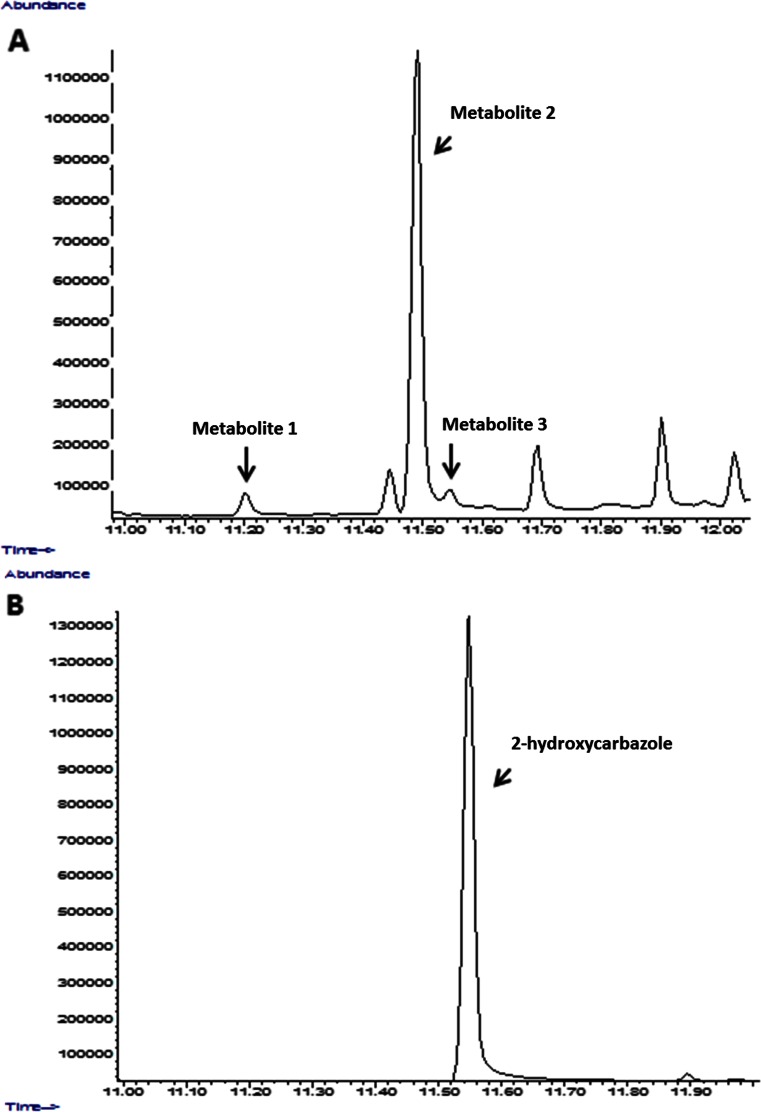
GC-MS chromatogram showing the carbazole derivatives with the retention times (*R*_t_) 11.2 min (metabolite 1), 11.49 min (metabolite 2), and 11.54 min (metabolite 3) formed by IM 1785/21Gp strain after 120 h incubation (**a**) and standard of 2-hydroksycarbazole (**b**)

**Fig. 4:**
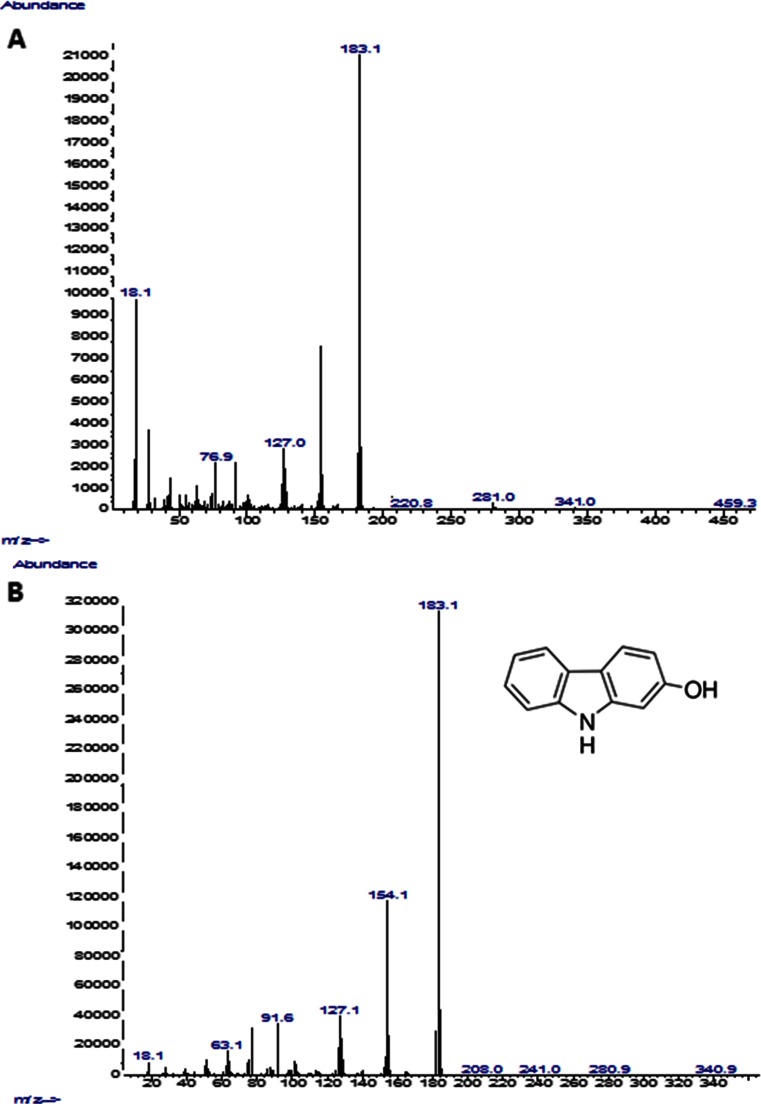
Mass spectrum of the carbazole derivatives with the retention times (*R*_t_) 11.54 min (metabolite 3) formed by IM 1785/21Gp strain after 120 h incubation (**a**) and standard of 2-hydroksycarbazole (**b**)

The examples of the chromatogram and mass spectra of carbazole products formed by *C. elegans* IM1785/21Gp are shown in Figs. 3a and 4a. The three carbazole derivatives were detected by qualitative and quantitative GC-MS methods. Three hydroxylated metabolites of carbazole with the retention times (*R*_t_) 11.2 min (metabolite 1), 11.49 min (metabolite 2), and 11.54 min (metabolite 3) min were detected and identified by a mass fragmentation pattern compared with NIST Standard Reference Database in the extracts of the IM 1785/21Gp and IM 2611 strains. In the case of the DSM 8217 strain, only metabolite 2 was detected. The monohydroxylated metabolites of carbazole were identified on the basis of a molecular ion at 183 m/z. Based on the comparison of the obtained chromatograms and mass spectra of carbazole metabolites produced by the tested microorganisms and available hydroxycarbazole standards, metabolite 3 was recognized as 2-hydroxycarbazole (Figs. 3b and 4b). For the first time, we identified 2-hydroxycarbazol as a product of carbazole biotransformation by the filamentous fungi of the *Cunninghamella.* In the case of metabolite 1 (*R*_t_ = 11.2 min) and metabolite 2 (*R*_t_ = 11.49 min), oxidation of carbazole in positions 2 and 4 was excluded, because the retention times of 2-hydroxycarbazole and 4-hydroxycarbazol were 11.54 and 11.41 min, respectively. Based on the comparison of our results with mass spectra and retention times available in literature (Yang and Davis 1992; Lobastova et al. 2004), it can be suggested that metabolite 2 is a hydroxylated product of carbazole in position 3. In the case of genus *Cunninghamella*, hydroxylated products were noted during the conversion of *N*-methylcarbazole. The fungus *C. echinulata* ATCC 9244 was shown to transform *N*-methylcarbazole to carbazole, *N*-hydroxymethylcarbazole, 3-hydroxycarbazole, and *N*-hydroxy-*N*-methylcarbazole (Yang and Davis 1992). The strain of *C. elegans* converted carbazole to 3-hydroxycarbazole (Lobastova et al. 2004; Parshikov et al. 2012). Lobastova et al. (2004) showed biotransformation of carbazole by *Aspergillus flavus* VKM F-1024 and detected three monohydroxylated metabolites, which were identified as 1-, 2-, 3-hydroxycarbazole. In a culture pre-incubated with 1-benzylindol, mono- and dihydroxylated derivatives of carbazole were identified. 2-Hydroxycarbazole was detected solely as an intermediate of carbazole transformation by *A. flavus* (Lobastova et al. 2004).

### Toxicity assay of carbazole metabolites

Carbazole is a well-known environmental pollutant which has a negative influence on different organisms. Sverdrup et al. (2003) showed high toxicity of carbazole toward *Trifolium pretense*, *Lolium perenne*, and *Sinepsis alba*. The 20 % reduction of seeding fresh weight (EC20-value) was evaluated in the range of 36–290 mg kg^−1^ (Sverdrup et al. 2003). The determination of the carbazole influence on *Danio rerio* showed its high hazardous potential. Carbazole was one of the most toxic NSO-heterocycylic compounds with LC50 1.07 mg L^−1^ (Peddinghaus et al. 2012). EC50-values of carbazole for *Daphnia* and *Vibrio fisheri* were 3.4 and 11.57 mg L^−1^, respectively (Blum et al. 2011). Moreover, the dioxin-like toxicity of carbazole was documented. Mumbo et al. (2015) showed the affinity of carbazole to the aryl hydrocarbon receptor (AHR). Nebert and Dalton (2006) supposed that activation of AHR by three polychlorinated biphenyl can result in the induction of cytochrome P450.

Metabolites of the hazardous pollutant formed during microbial degradation can be more harmful to living organisms than the parent compound (Bernat et al. 2013). Therefore, we analyzed the toxicity of the postculture extracts of the studied fungal strains. Artoxkit M was used for assessing the mortality of *A. franciscana* after the exposure to postculture extracts. The 48h LC50 value obtained for pure carbazole was 20.83 ± 3.82 mg L^−1^. The 48h LC50 values received for postculture extracts were similar to the value reached for carbazole. Metabolites formed by the tested fungi did not show higher toxicity than parent substrates. Similar results were obtained by Słaba et al. (2013) who did not observe an increase in the toxicity of alachlor and its derivatives. The authors suggested that alachlor degradation by *Paecilomyces marquandii* did not lead to the complete detoxification of the xenobiotic, but metabolites produced by *P. marquandii* can be more easily metabolized by other soil microorganisms.

### Changes in the fungi phospholipid composition in response to carbazole

The phospholipid (PL) profiles of the tested filamentous fungi were evaluated according to Bernat et al. (2014). The 46 species of PLs including phosphatidic acid (PA), phosphatidylethanolamine (PE), phosphatidylserine (PS), phosphatidylcholine (PC), and phosphatidylinositol (PI) were analyzed using the LC-MS/MS techniques. Recently, the modification of the microorganism’s lipid profile in response to the toxic compound has been shown (Dercová et al. 2004; Bernat et al. 2014; Xia and Yuan 2009). The dominant type of PLs in each filamentous fungus was PC and it comprised 48–59 % of the total cell PLs in samples without the addition of carbazole (Table 1). Our results are similar to those obtained by Bernat et al. (2013) who described the induction of oxidative stress and modification of the lipid profile of the *C. elegans* membrane by tributyltin. The values of 27–37, 6–12, 1.6–4.5, and 2–7 % were achieved for PE, PS, PI, and PA, respectively. Significant modification of PE contents were noted for IM 1785/21Gp and IM 2611 strains incubated with carbazole. The 6.3 and 4.7 % decrease in PE levels were observed in cells of IM 1785/21Gp and IM 2611 strains, respectively. The PC/PE ratio decides about the integrity of the membrane and the functioning of cells (Welti et al. 2002; Bernat et al. 2014). The differences in the PC/PE ratios after the incubation of the fungi with carbazole were determined. For the 1785/21Gp and IM 2611 strains, the PC/PE ratio increased by 28.6 and 18.9 %, respectively. The more significant enhancement of the PC/PE ratio and 30 % reduction in growth were noted in the cultures of the IM 1785/21Gp, which can suggest that among the three tested strains, IM 1785/21Gp is the most sensitive to carbazole. Moreover, there was a significant decline in the PS level observed in IM 1785/21Gp and IM 2611 cultures, which could have been caused by use of PS as a precursor in the PE and PC synthesis (Xia and Yuan 2009). Interestingly, an almost 1.5-fold significant increase in the PA level was noted in IM 1785/21Gp cells exposed to carbazole compared to control samples. PA is considered to be a signal lipid in response to many abiotic and biotic stress factors. An increase in the PA content in plants after the contact with salts, heavy metals, H_2_O_2_, and different pathogens was described (Testerink and Munnik 2005; Darwish et al. 2009). The significant growth of signal lipid level in IM 1785/21Gp cells was correlated with growth inhibition of this strain. Similar results were obtained for *C. elegans* incubated with tributyltin. It can be suggested that the growth of the PA level could have caused the decline in the level of PE, which can be used as a substrate for PA synthesis (Bernat et al. 2014). The significant increase of PI level was observed for IM 261 strain. PI play many important roles in eukaryotic cells. PI and its derivatives can stabilize cell membrane. Moreover, literature data suggest that PI can be involved in response of microorganisms to growth conditions (Gardocki et al. 2005). Increase of PI level in IM 2611 cells can be connected with high tolerance of this strain to tested xenobiotic. For the DSM 8217 strains, modification of phospholipid profile was insignificant what indicate that carbazole is the least toxic for this strain.

**Table 1:** The phospholipid composition [%] of the tested microorganisms from the stationary phase incubated with or without carbazole

Lipid species	IM 1785/21Gp		IM 2611		DSM 8217	
	Control	Carbazole	Control	Carbazole	Control	Carbazole
PA	4.63 ± 0.62	7.22 ± 1.83*	2.29 ± 0.27	3.26 ± 1.40	1.99 ± 0.59	3.10 ± 0.97
PE	33.99 ± 1.25	27.69 ± 3.47*	27.29 ± 0.41	22.59 ± 1.94*	37.84 ± 1.74	35.98 ± 4.37
PS	8.42 ± 1.40	6.26 ± 0.68*	9.84 ± 0.79	11.47 ± 0.29*	12.02 ± 1.11	11.93 ± 2.52
PC	48.88 ± 3.24	54.29 ± 4.12	58.50 ± 1.55	59.68 ± 0.77	46.49 ± 0.64	48.99 ± 5.36
PI	4.01 ± 0.39	4.54 ± 0.52	2.05 ± 0.07	3.14 ± 0.10*	1.64 ± 0.39	2.56 ± 0.73
PC/PE	1.44	1.96	2.14	2.64	1.23	1.36

Each result represents an average and SD taken from *n* ≥ 3. Statistical analysis was done using the Student’s test with *p < 0.005

## Conclusions

In the present study, the ability of the filamentous fungi *C. elegans* IM 1785/21Gp, *C. echinulata* IM 2611, and *C. elegans* DSM 8217 to utilize carbazole was determined. The GC-MS analysis of postculture extracts resulted in the identification of three carbazole derivatives. The pathway of carbazole degradation by the filamentous fungi of the *Cunninghamella* species involved the oxidation reaction. All tested microorganisms showed a capability of xenobiotic elimination. The IM 1785/21Gp and IM 2611 strains appeared to be very effective and produced three monohydroxylated derivatives of carbazole. In the cultures of these strains, about 90 % loss of the substrate was noted. DSM 8217 strain utilized 40 % of carbazole and formed one hydroxylated metabolite. 2-Hydroxycarbazole was identified for the first time as a carbazole metabolite formed by the filamentous fungi of the *Cunninghamella* species. Toxicity of carbazole and its metabolites toward *A. franciscana* was assessed. An increase in the mortality of larvae after the exposure to postculture extracts was not observed. Moreover, the influence of carbazole on the phospholipid profile of filamentous fungi was evaluated for the first time. The changes in the phospholipid levels after the exposure to carbazole suggested its highest toxicity to the IM 1785/21Gp strain and less harmful activity toward the DSM 8217 strain. The mechanism of the carbazole influence on the changes in the membrane phospholipid composition will be the subject of study in the future.
